# Mistakes

**Published:** 1889-04

**Authors:** WM. N. Morrison

**Affiliations:** St. Louis, Mo.


### Mistakes.

BY WM. N. MORRISON, D.D.S., ST. LOUIS, MO.

Read before the Mississippi Valley Dental Society, Cincinnati, March 7th, 1889.

While busy at the chair, a patient rushes in with a necessity-knows-no-law kind of a pace. Wants a tooth extracted, has not slept a wink all night, etc. Will hear no argument for its repair. He has tried the treatment you propose before, and the tooth had to be extracted after all. He then makes the time-honored remark which always brings the dentist up to the mark, "If you don't extract it I will go to somebody who will." It has ached constantly for a week, and he wants to be revenged. "As I was eating breakfast this morning," he says, "a little piece of bread got into the cavity and it has been on the jump ever since."

You are pretty sure to make a mistake what ever you do for such a patient. If you take him at his word, you make a mistake from which he is the victim the remainder of his life.

If you persuade him to have it properly treated and filled, and promise to keep him free from pain with it as long as he is within your reach, you are pretty sure to err again. Or he will have a putrescent pulp ripe to be drilled into the day you are in the country, or attending some dental meeting.

Other blunders one frequently falls into are:

Cutting away too much tooth substance which should be left for natural wear or breakage.

Cutting off weak crowns for more conspicuous metal substitutes.

Drilling through the apical foramen for the treatment of abscess, when we know there must ever after be an uncertainty about filling that canal.

Excavating soft dentine too close to the pulp, cleaning out layer after layer because it cuts easily and gives no pain.

Filling a cavity with one substance when another was more plainly demanded.

To call a fistulous opening through the alveolus a dangerous cancer, and frighten the patient into a spasm with "a-l-v-e-o-l-a-r a-b-s-c-e-s-s," uttered with French gymnastics and wise looks.

It has been my custom for years to saturate the spongy dentine of cavities with creosote, but I did not know all I was doing by the operation. I thought a carbolate of albumen and an arrest of ferment was enough.

But after witnessing Prof. Andrews' beautiful microscopical work on the screen, I am perfectly amazed at the slaughter of the innocents I have been committing. He demonstrated how the tubuli was entered by animalculi and enlarged until the whole structure was destroyed.

It is very plain that with a few of these tubuli so enlarged directly to the pulp, capillary action by compression or suction must be very direct. The too common mistake is to devitalize the pulp.

Let us watch keenly our own as well as the mistakes of others, for there is a valuable lesson in every one."

If Nature tolerates such enormous blunders and accepts the services of such bunglers, how much more comfortable she will be under the hand of skillful artists, alive with physological and pathological anatomy, microscopy, and a desire to accomplish the greatest good.

